# Effectiveness of methotrexate and leflunomide as corticoid-sparing drugs in patients with polymyalgia rheumatica

**DOI:** 10.1093/rap/rkae033

**Published:** 2024-03-21

**Authors:** Juan Pablo Vinicki, Alejandra Cusa, Daniela Domingo, José Luis Velasco Zamora, Sebastián Magri, Alejandro Brigante, Maria Marcela Schmid, Paola Ávila, Natalia Zamora, Laura Sorrentino, Anabella M Rodriguez, Miguel Linarez, Cecilia Pisoni, Carolina Costi, Gustavo Rodriguez Gil, María Andrea Spinetto, Vanesa Ursula Paris, Natalia Perrotta, María del Rosario Maliandi, Oscar Rillo, Claudia Pena, Julio Got, Javier Cavallasca, Maximiliano Machado Escobar, Carolina Iturralde, María Victoria Martire, Romina Tessel, N Saravia Chocobar, Graciela Alarcon

**Affiliations:** Sección Reumatología, Hospital de Quilmes, Buenos Aires, Argentina; IARI, Buenos Aires, Argentina; HIGA Luisa Cravenna de Gandulfo, Buenos Aires, Argentina; Instituto de Investigaciones Clínicas de Quilmes, Buenos Aires, Argentina; Unidad de Reumatología, Hospital Italiano de La Plata, Buenos Aires, Argentina; Servicio de Reumatología, Sanatorio Güemes, Buenos Aires, Argentina; Sección Reumatología, Hospital Llano, Corrientes, Argentina; Sección Reumatología, Hospital de Quilmes, Buenos Aires, Argentina; Sección Reumatología, HIGA San José, Buenos Aires, Argentina; Servicio de Reumatología, Sanatorio Güemes, Buenos Aires, Argentina; CEMIC, Sección Reumatología e Inmunología, Buenos Aires, Argentina; OSPM, Unidad de Reumatología, Buenos Aires, Argentina; CEMIC, Sección Reumatología e Inmunología, Buenos Aires, Argentina; Servicio de Reumatología, HIGA San Martín de La Plata, Buenos Aires, Argentina; Unidad de Reumatología, Hospítal Municipal Dr Leónidas Lucero, Buenos Aires, Argentina; Centro Médico Privado, Buenos Aires, Argentina; Unidad de Reumatología, Hospital Escuela de Agudos Dr Ramón Madariaga, Misiones, Argentina; Servicio de Reumatología, Hospital Dr César Milstein, Buenos Aires, Argentina; Unidad de Reumatología, Sanatorio Garay, Santa Fe, Argentina; Sección Reumatología, Hospital Ignacio Pirovano, Buenos Aires, Argentina; Servicio de Reumatología, HIGA San Martín de La Plata, Buenos Aires, Argentina; Unidad de Reumatología, Instituto Médico HUMANITAS, Chaco, Argentina; Sección Reumatología, Hospital José Bernardo Iturraspe, Santa Fe, Argentina; Sección Reumatología, Hospital Eva Perón, Tucumán, Argentina; Servicio de Reumatología, HIGA Dr Oscar Alende, Mar del Plata, Argentina; Servicio de Reumatología, HIGA San Roque de Gonnet de La Plata, Buenos Aires, Argentina; Servicio de Reumatología, Hospital Señor del Milagro, Salta, Argentina; Sección Reumatología, Hospital de Quilmes, Buenos Aires, Argentina; Division of Clinical Immunology and Rheumatology, Department of Medicine, Marnix E. Heersink School of Medicine, The University of Alabama at Birmingham, United States

**Keywords:** polymyalgia rheumatica, methotrexate, leflunomide, glucocorticoids, remission, relapse, recurrence, glucocorticoid-sparing drugs, observational study

## Abstract

**Objectives:**

The need for glucocorticoid-sparing drugs (GCSD) remains an important issue and is an unmet need in the treatment of polymyalgia rheumatica (PMR). We therefore aimed to assess the effectiveness and safety of methotrexate (MTX) and of leflunomide (LEF) in daily clinical practice in PMR patients from Argentina.

**Methods:**

A multicentre and observational study (medical records review) of PMR patients seen between 2007 and 2023, who had at least three months of follow-up after starting a GCSD, either MTX or LEF, was performed. Results are expressed as medians and interquartile ranges [25th–75th (IQR)] for continuous variables and percentages for categorical ones. The two treatment groups were compared using χ^2^ test for categorical variables, Mann–Whitney U test for continuous variables and the log-rank test for time-to-event data. Crude and adjusted odds ratios (ORs) with 95% confidence intervals (CIs) were calculated using logistic regression. In all cases, a *p*-value <0.05 was considered statistically significant.

**Results:**

One-hundred and eighty-six patients (79% female) with a median age of 72 years (IQR, 65–77 years) were included. One-hundred and forty-three patients (77%) were prescribed MTX (15, IQR 10–15) and 43 (23%) LEF (20 mg, fixed dose). Flare-ups (relapses and recurrences) occurred in 13 patients (7%) and were comparable between both groups. Persistent GCSD intake was observed in 145 patients (78%). Glucocorticoid (GC) withdrawal was achieved in 67 of these 145 patients (46%) and this occurred more frequently in the LEF group (*P = 0.001*). Furthermore, time until prednisone discontinuation was shorter in the LEF-treated patients (4.7 months, IQR 3–20 on LEF versus 31.8 months, IQR 10–82 on MTX, *P = 0.000*). Remission was found more frequently in the LEF group (*P = 0.003*). In the multivariate analysis, the probability of remission was higher with LEF therapy (*P *=* *0.010) and this finding persisted in the subgroup analysis who were followed up < 40 months (OR 3.12, 95% CI = 1.30–7.47, *P *= 0.011).

**Conclusions:**

This study demonstrated the clinical effectiveness of LEF and even its superiority in achieving remission when compared with MTX as GCSD in PMR patients. Further research is needed to support these findings.

Key message

- LEF could be an option in PMR patients with risk factors for relapse and/or prolonged GC therapy but who have suffered an adverse effect or have a contraindication to MTX.

## Introduction

Polymyalgia rheumatica (PMR) is a condition that affects older people and is characterized by proximal muscle pain and stiffness [1]. The mainstay of therapy is oral glucocorticoids (GC). However, up to 60% of patients experience disease relapses during GC tapering, and several studies indicate that GC can rarely be discontinued before two years [2].

The EULAR/ACR panel conditionally recommends methotrexate (MTX) for PMR in patients with risk factors, comorbidities, concomitant medications where GC-related adverse events are more likely to occur, experiencing GC-related adverse events or who have relapsed (either on or off GCs) [3]. A stronger recommendation is not supported and this is due to the fact that the total number of patients included in randomized trials has been small and the results have been contradictory; the later may be due, in part, to the very low quality of the evidence on the negative trials. Lastly, a reduction in GC-related adverse events with the use of MTX has not been demonstrated.

Recently, we have examined the risk factors for relapse and/or prolonged GC therapy in patients with PMR with the aim of identifying which patient could benefit from the early introduction of a GC-sparing drug (GCSD) [4]. We are now assessing the effectiveness and safety of MTX and leflunomide (LEF) in daily clinical practice in PMR patients from Argentina.

## Materials and methods

A multicentre and observational study (medical records review) of PMR patients diagnosed according to the Chuang criteria [5] seen at the outpatient clinics of 23 public or private rheumatology units in Argentina, between 2007 and 2023, and who had at least a 3-month follow-up period after starting a GCSD, was performed. Data were obtained from the patients’ medical records and stored in a computerized database. Giant cell arteritis (GCA) at diagnosis or during follow-up was considered an exclusion criterion. The study was conducted in compliance with the Declaration of Helsinki, International Conference on Harmonization Guidelines for Good Clinical Practice, and local country regulations. The final protocol and amendments were approved by the local ethics committee (Instituto de Efectividad Clínica de Quilmes, Buenos Aires, Argentina). The participants’ written consent was not required according to local legislation (Resolución 1480/2011, Ministerio de Salud de la Nación).

The data obtained at initiation of GC and GCSD therapy included gender, age, associated comorbidities (on the basis of medical diagnosis noted in the patient’s medical records), disease diagnosis date, clinical presentation (pain and/or stiffness in shoulder girdle, pelvic girdle, shoulder girdle and pelvic girdle, associated arthritis) and laboratory markers when available (erythrocyte sedimentation [ESR], C-reactive protein [CRP]). Regarding GC therapy, the data included dose at PMR diagnosis, at baseline, date of GC discontinuation (if applicable) and minimum dose required to avoid relapses (if applicable). GC starting dose and weaning occurred at the discretion of the treating physician. Data of GCSD included type of drug (MTX, LEF) and dose, date of initiation, date of flare-ups, that is relapses or recurrences or both, (if applicable), date of discontinuation (if applicable) and reason (relapse, recurrence or adverse effect, AE). PMR patients with arthritis but who did not fulfil classification criteria for rheumatoid arthritis (RA) and who were not under immunosuppressive therapy were included.

A *flare-up* was defined as the presence of signs and symptoms of PMR (aching and stiffness on shoulder or hip girdles, or in both) accompanied by an increased ESR (> 30 mm/h), CRP (> 5 mg/l) or both; two types of flare-ups were considered: *relapses* and r*ecurrences. Relapse* was defined if these symptoms were observed during steroid tapering; *recurrence* when symptoms occurred after steroid withdrawal. Remission was defined as the absence of signs and symptoms of PMR accompanied by a normal ESR (< 30 mm/h) and/or CRP (<5 mg/l) and free of GC without recurrence. Patients reported AEs were based on a list of seven possible causes: lung toxicity, haematological toxicity, liver toxicity, gastrointestinal symptoms (oral ulcers, dyspepsia, nausea, vomiting, abdominal pain and diarrhea), alopecia, skin rashes and other.

## Statistical analysis

Results are expressed as medians and interquartile ranges [25th–75th (IQR) for continuous variables and percentages for categorical ones. The two treatment groups, MTX and LEF, were compared using χ^2^ test for categorical variables, Mann–Whitney U test for continuous variables and the log-rank test for time-to-event data. Crude and adjusted odds ratios (ORs) with 95% confidence intervals (CIs) were calculated using logistic regression. Adjusted OR were calculated adding each variable manually one by one. The adjusted ORs were reported when the OR was modified by at least 10% after incorporating the variable into the model. In all cases, a *p*-value <0.05 was considered statistically significant. Statistical analyses were performed with Stata 14.0 (StataCorp, Texas, USA).

## Results

### Baseline characteristics

One-hundred and eighty-six patients were included (79% female) with a median age of 72 years (IQR, 65–77 years). Descriptive data for these patients are presented in Table 1. Relevant comorbidities included hypertension (60%), diabetes (17%), dyslipidaemia (25%) and history of smoking (9%). The most frequent clinical presentation was pain and/or stiffness in shoulder and pelvic girdles (61%), whereas arthritis occurred in 35% of the patients. Regarding therapy, prednisone at a median dose of 15 mg (IQR 10–20) was prescribed at the time of PMR diagnosis.

**Table 1.** Clinical characteristics and univariate comparison of patients with polymyalgia rheumatica at diagnosis and at baseline of the corticosteroid-sparing drug

	Total n = 186	MTX n = 143	LEF n = 43	*P* value
Female gender, n (%)	147 (79)	114 (79)	33 (77)	0,674
Age in years, median (IQR)	72 (65–77)	73 (66–78)	69 (61–76)	*0,031*
Comorbidities, n (%)				
Hypertension	112 (60.2)	87 (60.8)	25 (58.1)	0.751
Diabetes	31 (16.7)	24 (16.7)	7 (16.3)	0.938
Dyslipidaemia	47 (25.3)	32 (22.4)	15 (34.9)	0.098
Smoking history	16 (8.6)	13 (9)	3 (7)	0.665
Hypothyroidism	47 (25.3)	37 (26.9)	10 (23.3)	0.729
Clinical presentation at diagnosis, n (%)				
Pain and/or stiffness in				
shoulder girdle	75 (40.3)	65 (45.5)	10 (23.3)	*0,009*
pelvic girdle	38 (20.4)	32 (22)	6 (14)	0,230
shoulder girdle and pelvic girdle	114 (61.3)	85 (59.5)	29 (67.5)	0,345
Arthritis	65 (35)	53 (37)	12 (28)	0.270
Laboratory, median (IQR)				
At diagnosis				
ESR	55 (30-81)	56 (32-85)	43 (14-71)	*0,014*
CRP	14 (8.6-40)	16 (10-47)	6 (0-18)	*0,0001*
At baseline				
ESR	30 (17-45)	30 (19-48)	22 (12-38)	*0,0094*
CRP	6 (1-12)	6 (3-12)	0.26 (0-8)	*0,0000*
Dose of GC (mg), median (IQR)				
At diagnosis	15 (10-20)	15 (10-20)	20 (10-20)	0,844
At baseline	10 (6-12.5)	10 (7.5-15)	10 (5-10)	*0.006*
Disease duration before GCSD (mo), median (IQR)	4.4 (1.6-10)	4.3 (1.4-10.3)	4.6 (1.8-10)	0.645

ESR: erythrocyte sedimentation; CRP: C-reactive protein; MTX: methotrexate; LEF: leflunomide; wk: weeks; GCSD: glucocorticoid-sparing drug; GC: glucocorticoid; mo: months.

As to treatment, 77% (*n* = 143) were prescribed MTX (15 mg, IQR 10–15) and 23% (*n* = 43) LEF (20 mg, fixed dose) as GCSD (Table 1). At baseline, gender and comorbidities were comparable between these two groups; however, patients on LEF therapy were younger than those on MTX (69 years, IQR 61–76 for LEF versus 73 years, IQR 66–78 for MTX, *P = 0.031*). No differences in the clinical presentation at diagnosis were found between these groups except for pain and/or stiffness in the shoulder girdle (45% on MTX versus 23% on LEF, *P = 0.009*). Higher ESR and CRP levels were observed in the MTX group, at diagnosis and at baseline with a statistically significant difference as noted in Table 1. Furthermore, the dose of GC at baseline visit was slightly higher in the MTX than in the LEF group with a statistically significant difference (10 mg, IQR 7.5–15 on MTX versus 10 mg IQR 5–10 on LEF, *P = 0.006*). Lastly, no difference in disease duration before the initiation of GCSD was observed between groups (4.3 months IQR 1.4–10.3 on MTX versus 4.6 months IQR 1.8–10 on LEF, *P *= 0.645).

### Follow-up, flare-ups and comparison between groups. Univariate analysis

After therapy began, flare-ups occurred in 13 of 186 patients (7%) with no difference between groups (Table 2*, Section A*); that was the case for both types of flare-ups, relapses and recurrences. In patients who were unable to stop GC despite the administration of a GCSD, no differences in GC dose required to avoid relapse (5 mg, IQR 2.5–6.25 with MTX versus 5 mg, IQR 4–7.5 with LEF, *P *= 0.729) was observed. The same was the case for the GC-sparing effect (5 mg, IQR 0–10 on MTX versus 5 mg IQR 0–7.5 on LEF, *P *= 0.814).

**Table 2.** Univariate comparison throughout the follow-up period between MTX and LEF therapy. Section B includes fewer patients (n = 145) due to corticosteroid-sparing drug discontinuation (n = 41)

A	Total n = 186	MTX n = 143	LEF n = 43	*P* value
Flare-up, n (%)	13 (7)	8 (6)	5 (11.6)	0.182
Relapse, n (%)	4 (2)	2 (1.4)	2 (4.6)	0.333
GCSD withdrawal, n (%)	41 (22)	30 (21)	11 (25)	0.523
Time to GCSD withdrawal, (months), median (IQR)	3.75 (2–10.3)	3.91 (2–10.7)	3.14 (2–7.3)	0.626

B	Total n = 145	MTX n = 113	LEF n = 32	*P* value
Laboratory, median (IQR)				
At month 3				
ESR	20 (12–32)	21.5 (14–32.5)	15 (10–21)	*0.005*
CRP	5 (0.5–6.6)	5 (1–6.9)	1 (0–6)	*0.0109*
Δ from baseline to month 3				
ESR	10 (2–19)	10 (3–23)	6 (0–17)	0.210
CRP	2 (0–7)	2.75 (0–8)	0 (0–3.4)	0.085
Time after the initiation of GCSD to the last observation (months), median (IQR)	14 (5–26)	16.8 (5.4–30.7)	8 (3.9–18.2)	*0.0222*
GC withdrawal, n (%)	67(46)	44 (39)	23 (72)	*0,001*
Recurrence, n (%)	9 (13)	6 (13)	3 (13)	0.329
Remission, n (%)	58 (40)	38 (33.6)	20 (62.5)	*0.003*

MTX: methotrexate; LEF: leflunomide; GC: glucocorticoid; GCSD: glucocorticoid-sparing drug.

Δ: difference.

**Figure 1.**
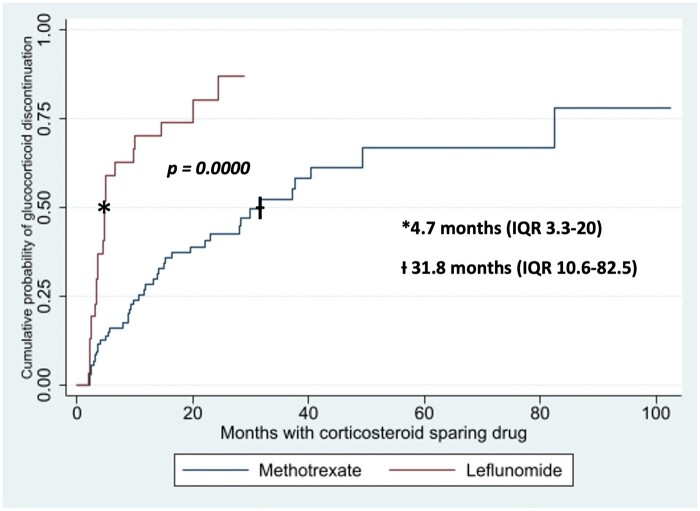
Kaplan-Meier curve of time to prednisone discontinuation (*n* = 145)

Regarding GCSD discontinuation (*n* = 41), there were no differences between groups. Likewise, persistence (*n* = 145) was observed with comparable frequency in both groups (79% on MTX versus 74% on LEF, *P *=* *0.523); however, the time after the initiation of GCSD to the last observation was significantly longer in the MTX group (16.8 months IQR 5–31) versus 8 months IQR 4–18 on LEF group, *P = 0.022*); higher ESR and CRP levels were observed in the MTX group at month 3 with a statistically significant difference (Table 2*, Section B*). GC withdrawal was achieved in 67 of 145 patients (46%) and this goal occurred more frequently on the LEF group (39% on MTX versus 72% on LEF, *P = 0.001*). Furthermore, time until prednisone discontinuation was shorter in the LEF-treated patients (32 months IQR 10–82 with MTX versus 5 months IQR 3–20 on the LEF-treated, *P = 0.000*). These data are shown in Fig. 1. Remission was found more frequently in the LEF-treated group (33.6% versus 62.5%, *P = 0.003*). No significant differences between groups regarding recurrences were found. However, ≥ 2 recurrences occurred only in the MTX group. Regarding MTX dose at baseline and GC discontinuation during follow-up, we found a higher proportion of patients that achieved this goal when the MTX dose was > 15 mg per week (*P = 0.009*, Supplementary Table S1, available at *Rheumatology Advances in Practice* online).

### Remission

When PMR patients in and not in remission were compared by bivariate analysis, the group not in remission had an increased frequency of pain and/or stiffness in the shoulder girdle (36% in remission versus 53% not in remission, *P *=* *0.049). As previously described, remission occurred more frequently on the LEF group (OR = 3.29; 95%CI = 1.46–7.43; *P = 0.004*), but no other significant differences between groups were found (Supplementary Table S2, *available at Rheumatology Advances in Practice* online). The only variable that changed the LEF therapy OR more than 10% in multivariate analysis was pain and/or stiffness in the shoulder girdle. This model showed an increase in the probability of remission with LEF therapy (LEF therapy [OR] 2.96, 95% CI = 1.29–6.78, *P *= 0.010; pain and/or stiffness in the shoulder girdle [OR] 0.59, 95% CI = 0.29–1.19, *P *= 0.141). According to Table 2B, time after the initiation of GCSD to the last observation was significantly longer in the MTX group; therefore, a subgroup analysis was performed that included 93 patients from the MTX group and 31 patients from the LEF group who were followed up < 40 months (maximum follow-up time interval of the LEF group); the results in the model were similar (LEF therapy: OR 3.12, 95% CI = 1.30–7.47, *P *=* *0.011; pain and/or stiffness in the shoulder girdle: OR 0.48, 95% CI = 0.22–1.04, *P *=* *0.063).

### Adverse effects

Forty-one patients discontinued the GCSD (4 cases with ≥ 2 relapses that led to drug withdrawal without difference between groups) but 37 suffered an AE. The most frequently reported AE was gastrointestinal symptoms (oral ulcers, dyspepsia, nausea, vomiting, abdominal pain, diarrhea) in 17 patients (9%) without a significant difference between groups followed by haematological toxicity in 7 patients (4%) only on the MTX group and skin rash only on the LEF group (*P = 0.010*). These data are presented in Supplementary Table S3, available at *Rheumatology Advances in Practice* online.

## Discussion

Early introduction of a GCSD is best considered in PMR patients with comorbidities and/or concomitant medications where GC-related adverse events are more likely to occur. The need for a GCSD, either synthetic or biologic, remains an important issue and is an unmet need in the treatment of PMR. Moreover, finding a low-cost GCSD is extremely important because patient access to biologic therapy (like tocilizumab or sarilumab) is out of reach for many patients, especially in Latin America (LatAm).

Relapses and recurrences in PMR have been examined in previous studies. In the Lugo study from Northwestern Spain, for example, patients with isolated PMR (*n* = 134) treated only with GC and without GCSD had frequent relapses (23.1%) and much less recurrences (6.4%) [6]. In a study from Rochester, Minnesota, relapses (defined as an exacerbation of PMR symptoms, either on or off GCs, requiring an adjustment of GC dose) were found to occur in 55% of the patients from a population based cohort (*n* = 364) [7]. In a study from Buenos Aires, Argentina, of 185 PMR patients, 34.2% of them relapsed (as per the same definition as that of the Rochester study) [4]. In this regard, we should point out that in our study, the addition of MTX or LEF was followed by a significantly low frequency of disease exacerbations over time; after such therapy, flare-ups occurred in 17 of 186 patients (7%). The proportion of PMR patients that presented a flare-up, relapse or recurrence were comparable in the MTX- and LEF-treated groups. However, remission was found more frequently in the LEF group. To the best of our knowledge, this is the first study to assess effectiveness and safety of LEF in PMR patients from Latin America in daily clinical practice. Additionally, a comparative evaluation with MTX was considered useful since it is the recommended GCSD in the 2015 ACR/EULAR guidelines [3]. Moreover, the efficacy and safety of other conventional synthetic DMARDs is a question that deserves to be researched.

MTX is the most commonly used GCSD, at a starting dose of 7.5–10 mg per week. Studies of the effectiveness of MTX are conflicting, some show no benefit, while others show benefit, suggesting that MTX is useful to achieve remission, and to reduce the number of relapses, although overall the reported benefits are generally small [8–10]. Available data from randomized controlled studies are limited by several factors including the small number of patients included, the relatively low doses of MTX used and a high dropout rate. A limitation of the current study is that we considered the starting dose of MTX but not if, during follow-up, adjustment of such baseline dose was required; this is highly likely, especially in those patients with a chronic relapsing phenotype. The use of MTX in doses currently used for the management of RA (up to 25 mg/week) has not been formally studied in PMR. Interestingly, we found that when MTX dose was > 15 mg per week, GC discontinuation was achieved more frequently. This is a potential area for future research.

Regarding LEF, data are too scarce to allow generalizations. These data come from two case series of difficult-to-treat PMR patients in whom time to response was achieved in two to three months; in addition, CRP reduction and a significant reduction in steroid dosage was observed in almost all cases [11, 12]. It seems that the active metabolite of LEF, A77 1726, interferes with dendritic cell function and impairs some events that involve the JAK/STAT pathway and control proinflammatory TNF and IL-17 cytokine production which are all involved in the pathogenesis of GCA and PMR [13–15]. Also, LEF modulates IL-6 levels, known to be elevated in PMR and GCA [16]. Interestingly, we found that GC withdrawal was achieved more frequently within the LEF group. Moreover, time until GC discontinuation was shorter in the LEF (median 5 months, IQR 3–20) than in the MTX group (median 32 months, IQR 10–82). These findings are quite similar to the ‘time to response’ reported in case series but we prefer to be cautious due the nature of our study. GC at baseline and tapering occurred at the discretion of the treating physician and were not preset. We consider that this variability depends on the physicians’ medical training and clinical experience and on the patient profile; these considerations might have played a role, therefore, we cannot rule out if this group has undergone a rapid decrease in GC dose. As well, we cannot state whether every physician on our study prescribed an initial starting dose or approached weaning according to the 2015 EULAR/ACR guidelines’ recommendations. Nevertheless, remission was found more frequently in the LEF group, but no significant differences were found between groups regarding recurrences. Although long-term follow-up differed between groups, an increased probability of remission with LEF therapy was still found in the subgroup analysis.

Raised values of ESR and CRP are typical in patients with PMR at the time of diagnosis. The range of the CRP in PMR, like the ESR, is wide. PMR with low ESR is considered a more benign form of disease, with a lower frequency of constitutional manifestations compared with PMR with a high ESR [17]. Although differences in ESR and CRP between both groups at the time of recruitment might suggest that patients treated with MTX had more severe disease because they had a greater initial inflammatory burden, median ESR and CRP in both groups were still above the upper limit of normal and there were no meaningful differences in clinical presentation aside from the degree of pain and/or stiffness at the shoulder girdle (which does not imply a more severe disease phenotype).

This study has several limitations. First, data from some patients were missing (historical clinical data). Secondly, sampling of the centres has not been random (availability of the records), and this may pose a selection and information bias in the results (for example: higher ESR/CRP at diagnosis and baseline, higher GC dose at baseline, GC weaning, MTX dose at baseline). Thirdly, there is a lack of long-term follow-up, which was particularly the case in the LEF group. Despite these limitations and to our knowledge, there are no studies from Latin America examining MTX and LEF as GCSD in PMR patients. Also, this study demonstrated the clinical effectiveness of LEF compared with MTX and the achievement of remission more likely to be accomplished with the former. Further research is needed to support these findings.

## Supplementary Material

rkae033_Supplementary_Data

## Data Availability

The data underlying this article will be shared on reasonable request to the corresponding author.
