# Supplementary material for: Effectiveness of methotrexate and leflunomide as corticoid-sparing drugs in patients with polymyalgia rheumatica
Source: Rheumatol Adv Pract. 2024 Mar 21;8(2):rkae033. doi: 10.1093/rap/rkae033 (PMC10978571; doi:10.1093/rap/rkae033)
Supplement: rkae033_Supplementary_Data [file rkae033_supplementary_data.docx]

*Supplementary Table S1. Methotrexate (MTX) dose at baseline and glucocorticoid (GC) discontinuation*

*during follow-up (n = 113).*

| MTX dose (mg) | n | GC discontinued, n (%) |
| --- | --- | --- |
| 7.5 | 6 | 0 (0) |
| 10 | 38 | 14 (37) |
| 15 | 55 | 20 (36) |
| 20 | 9 | 5 (56) |
| 25 | 5 | 5 (100) |

*Note that higher proportion of patients that achieved this goal when MTX dose was >15 mg per week (p = 0.009).*

Supplementary Table S2. Bivariate analysis of PMR patients who did and did not achieved remission.

|  | REMISSION  n = 58 | NO REMISSION  n = 87 | *p* value |
| --- | --- | --- | --- |
| Female gender, n (%) | 44 (75.9) | 68 (78.1) | 0.746 |
| Age in years, median (IQR) | 69 (62-77) | 72 (66-77) | 0.299 |
| Comorbidities, n (%)  Hypertension  Diabetes  Dyslipidemia  Smoking history  Hypothyroidism | 32 (55)  12 (20.7)  16 (27.6)  9 (15.5)  20 (34.5) | 59 (67.8)  15 (17.2)  26 (29.9)  6 (7)  20 (23) | 0.123  0.601  0.765  0.095  0.129 |
| Clinical presentation at diagnosis, n (%)  Pain and/or stiffness in  shoulder girdle  pelvic girdle  shoulder girdle and pelvic girdle  Arthritis | 21 (36.2)  10 (17.2)  37 (63.8)  17 (29.3) | 46 (52.9)  26 (29.9)  47 (54)  35 (40.2) | *0.049*  0.084  0.243  0.179 |
| Laboratory, median (IQR)  At baseline  ESR  CRP  ∆ from baseline to month 3  ESR  CRP | 30 (17-47)  6 (0-12)  10 (4..5-21)  3.3 (0-8.6) | 32 (20-45)  6 (3.2-12)  10 (3-23)  2.75 (0-6) | 0.611  0.219  0.751  0.372 |
| Dose of GC (mg), median (IQR)  At baseline | 10 (5-12) | 10 (7.5-15) | 0.316 |
| Time of disease evolution (mo), median (IQR) | 4 (1.5-9.4) | 3.3 (1.4-10.2) | 0.816 |
| Corticosteroid-sparing drug, n (%)  Methotrexate  Leflunomide | 38 (33.6)  20 (62.5) | 75 (66.4)  12 (37.5) | *0.003* |

ESR = erythrocyte sedimentation; CRP = C-reactive protein; ∆ = difference; wk = weeks; GCSD = glucocorticoid-sparing drug; GC = glucocorticoid; mo = months.

*Supplementary Table S3. Reported adverse effects of the corticosteroid-sparing drug throughout the follow-up period.*

|  | Total  n = 186 | MTX  n = 143 | LEF  n = 43 | *p* value |
| --- | --- | --- | --- | --- |
| Lung toxicity, n (%) | 1 (0.7) | 1 (0.7) | 0 (0) | 0.769 |
| Haematological toxicity, n (%) | 7 (3.76) | 7 (4.9) | 0 (0) | 0.139 |
| Liver toxicity, n (%) | 2 (1.08) | 1 (0.7) | 1 (2.33) | 0.365 |
| Gastrointestinal symptoms (oral ulcers, dyspepsia, nausea, vomiting, abdominal pain, diarrhea) | 17 (9.14) | 14 (10) | 3 (7) | 0.575 |
| Alopecia, n (%) | 2 (1.08) | 1 (0.7) | 1 (2.33) | 0.365 |
| Skin rash, n (%) | 2 (1.08) | 0 (0) | 2 (4.65) | *0.010* |
| Other, n (%) | 7 (3.76) | 5 (3.5) | 2 (4.7) | 0.727 |
